# Conceptualizing callous-unemotional traits in preschoolers: Associations with social-emotional competencies and aggressive behavior

**DOI:** 10.1186/s13034-021-00376-4

**Published:** 2021-05-20

**Authors:** Jelena Zumbach, Annika Rademacher, Ute Koglin

**Affiliations:** 1 Family Law Psychology, Psychologische Hochschule Berlin, Am Koellnischen Park 2, 10179 Berlin, Germany; 2 Faculty of Educational and Social Sciences, Department of Pedagogic and Rehabilitation Psychology, University of Oldenburg, Ammerlaender Heerstr. 114118, 26111 Oldenburg, Germany

**Keywords:** Callous-unemotional traits, Aggressive behavior, Preschool, Psychopathy

## Abstract

**Background:**

Extensive empirical evidence suggests that high Callous-Unemotional (CU) traits in childhood and adolescence can reliably identify individuals at risk for antisocial outcomes. The present study addresses research gaps by investigating the factor structure of CU traits in children at preschool age.

**Methods:**

The sample includes 371 children (49.6% female, *M* age=4.7, *SD*=0.69). Using the Inventory of Callous-Unemotional-Traits (ICU), six alternative confirmatory factor analyses were conducted to find the best fitting model for our preschool sample. Childrens level of emotional competence and aggressive behavior was assed using a German questionnaire, the *Behavior Rating Scales for Preschoolers* (Verhaltensskalen fr das Kindergartenalter, VSK) in a preschool teachers rating. Post hoc cluster analytic strategies and ANOVA were applied to identify groups of children with regard to their combination of social-emotional competences and CU traits, and to examine associations with aggressive behavior.

**Results:**

Results indicate that a two-factor model revealed the best fit to our data, including a callous and an uncaring factor using 12 of the original 24 ICU items. Cluster analytic strategies reveal a risk group of children demonstrating high rates of callousness and uncaring combined with weak emotion knowledge/empathy and social competence. ANOVA shows that children in the risk group demonstrate the highest levels of aggressive behavior.

**Conclusions:**

Group characteristics indicate that the construct of CU traits in early childhood may be nothing other than a social-emotional developmental deficit.

## Introduction

Psychopathy describes a severe form of personality disorder in adults that is characterized by affective, interpersonal and behavioral features [1, 2]. Psychopathic traits refer to a subgroup of individuals who display massive antisocial behavior associated with severe forms of violence, aggression and criminal behavior [3, 4]. Those affected show a lack of empathy, a lack of feelings of guilt and social responsibility [4].

Research on psychopathy increasingly focuses on the occurrence and the development of early onsets in childhood and adolescence [5]. The term of callous-unemotional traits (CU traits) refers to characteristics, which are similar to the affective features of adult psychopathy [6]. Both, CU traits as well as adult psychopathy are characterized by a lack of remorse and guilt, impaired empathy and insensitivity to others feelings, a shallow or deficient affect, and unconcern about poor performance [7, 8, 21]. Extensive empirical evidence suggests that high CU traits in childhood and adolescence can reliably identify individuals at risk for severe antisocial outcomes [9,11].

Given the serious risk of CU traits for severe behavior problems over the course of life and moreover to examine the potential of early prevention, it is of great interest to detect symptoms as early as possible. Therefore, studies have increasingly examined the role of CU traits in young children and reported evidence that CU traits are already discriminatory at preschool age [12,14].

To asses CU traits in youth and adolescents, studies often use the *Inventory of Callous-Unemotional-Traits* (ICU; [15], e.g., [16,18]). The questionnaire was developed to provide means for assessing the callous and unemotional aspects of psychopathy. The ICU consists of parent- and teacher report versions to be used primarily with children and a self-report version for use with adolescents and young adults. Specific age-related versions do not exist. The ICU includes 24 items and comprises the three subscales of *callousness, uncaring* and *unemotional* [15]. The questionnaire exists as an approved German translation. Further details are presented in the methods section.

In recent years, the ICU has been increasingly used to measure CU traits in younger children (e.g., [11, 12, 19]. Studies consistently showed a relationship between CU traits and conduct problems [6, 12], Willougby et al. 2015). Associations with aggressive behavior are particularly emphasized in childhood and youth, with CU traits being a stable predictor of aggressive behavior [9, 20]. Accordingly, the presence of CU traits can designate a subgroup of children showing a severe risk for maladaptive development [21].

The factor structure of the ICU has been widely studied, but in many studies only on youth and adolescent samples (e.g., [18, 22, 23]). Although CU traits are often analyzed in childhood, factor-analytic studies involving younger children at preschool age are rare (e.g., [12, 13, 24]). Additionally, there are inconsistencies in the specification of the construct of CU traits using the ICU in samples of children and adolescents. A large number of ICU models exist in the literature that have been partially confirmed or rejected in various factor-analytical studies (e.g., [6, 25]).

Confirmatory factor analyses have been conducted by various studies without showing a clear consensus. Initial psychometric work on youth and adolescent samples supported a three-factor-bi-factor model including the three specific dimensions callousness, uncaring and unemotional, in combination with a general factor on which all items load simultaneously [17, 23, 26]. Following studies reported that this proposed factor structure fits their data rather poorly, even when similar age groups were considered. Ezpeleta and colleagues (2013) are one of the few who included children aged 4 to 6 years in their sample. The authors confirmed a three-factor structure with correlated factors. Ciucci et al. [16] found CU traits best described as three subfactors (callousness, uncaring, unemotional) with an overarching higher-order factor in a sample of children in grades 6 and 8.

Willoughby et al. [25] argue that items from the uncaring scale are ambiguously used, as they comprise reversed-scored items (e.g., Apologizes (says he/she is sorry) to persons he/she has hurtrecoded) to measure uncaring behavior. Thus, the authors argue that they rather represent the presence of empathic and prosocial behavior than a reverse measure of impaired empathy or insensitivity to others feelings. Confirmatory factor analysis indicated a two-factor solution with an empathic-prosocial and a callous-unemotional factor for school-aged children. Hawes and colleagues [6] used item response theory techniques to achieve a measure refinement on a sample of children at the age of 6 to 12 years. After eliminating 12 of the original 24 ICU items, a two-factor model with 12 remaining items was reported that distinguished callousness and uncaring behavior. Kimonis et al. [13] replicated that finding in a sample of 214 children at preschool age, reporting that confirmatory factor analyses supported a two-factor structure including callous and uncaring dimensions from 12 of the 24 original ICU items.

Similar results were found by Bansal et al. [24] who tested existing ICU models in a sample of 104 preschool children. The authors furthermore identified most central items via statistical examination of inter-item relationships / network analysis (Does not care who he / she hurts to get what they want , Does not care if he / she is in trouble , and Seems very cold and uncaring ).

The results from previous studies therefore show that it is important to specify a model before investigating relationships. Similarly, it cannot be assumed that models for older samples can be transferred to younger children without being tested.

Independent of the factor structure, study results consistently show associations between CU traits and conduct problems in samples of children and adolescents. This indicates that CU traits can predict negative developmental trajectories [6, 12], Willougby et al. 2015). On the other hand, it is a common consensus that social-emotional competencies positively influence childrens adaptive development [27]. Especially children with high levels of empathy understand and share the feelings of others and show less maladaptive and aggressive behavior [28].

Since high CU traits are associated with a lack of empathy, low emotional responsiveness and unconcern about others (e.g., [29]), the question arises whether CU traits and social-emotional competencies are two independent constructs or two poles of the same construct. The first case suggests that children can demonstrate high levels of both constructs. For example, this can be the case for children showing high levels of emotion knowledge and high abilities of self-regulation, but at the same time showing callous features, such as an insensitivity to others feelings and a lack of remorse and guilt. In this case, high social-emotional competencies combined with high CU traits could be seen as a protective factor. In the second case, high CU traits in childhood could be interpreted as a social-emotional development deficit.

Studies on CU traits and behavior problems in childhood often do not consider childrens social-emotional development in their analyses, which leads to a limited interpretation of results. Correlations with behavior problems, e.g., aggressive behavior are commonly investigated either for CU traits (e.g., [12]) or childrens level of social-emotional development (e.g., [30]). To the authors knowledge, only Kimonis et al. [13] evaluated the factor structure, psychometric properties, and validity of the ICU not only in relation to measures of antisocial/prosocial behavior, but also in relation to emotional processing. Findings indicate that preschool children high on CU traits were less accurate in recognizing facial expressions. Additionally, children were less attentionally engaged by images of others in distress, however only when children presented co-occurring conduct problems. Questions on whether childrens development of social emotional skills and childrens levels CU traits can each influence the emergence of conduct problems, or whether they represent two poles of the same construct, remain unanswered.

## The current study

CU traits are increasingly studied in childhood in association with behavioral problems (e.g., [31, 32]). However, there is a research gap concerning how CU traits in children at preschool age are best conceptualized. With this study, we aim to (a) analyze the factor structure of CU traits in a sample of preschool children, and to (b) identify groups of preschool children with regard to both CU traits and their social-emotional development to investigate associations with levels of aggressive behavior. We pursue to contribute to a better understanding of why some groups of children are at higher risk for a development of behavioral problems at preschool age, simultaneously taking CU traits and social-emotional development into account.

## Method

### Participants and procedure

The present study was conducted in Northern Germany. The data collection took place at preschool age where preschool teachers rated childrens level of CU traits and social-emotional competencies. The participating children were recruited from 39 preschools in Northern Germany (ad hoc sampling). All children were in their last year of preschool before school enrollment.

The sample includes *N*=371 children (49.60% female) with complete datasets with a mean age of *M*=72.44 months (*SD*=4.19; ranging from 62 to 88 months). For 21.70% of the children, an immigrant background is reported. Participation of the children and preschools was voluntary. The study was approved by the universitys ethics committee and received a positive vote from the national school authorities. Informed consent and written parental permission were obtained.

### Measures

*CU traits.* CU traits were measured at preschool age by applying the ICU (preschool version; [15]). Preschool teachers rated how well a statement describes a child on a four-level scale from "not at all true" (0) to "definitely true (3). The ICU questionnaire includes the three scales *callousness* (11 items, e.g., Shows no remorse when he/she has done something wrong , Does not care about doing things well), *uncaring* (8 items, e.g., Apologizes (says he/she is sorry) to persons he/she has hurtrecoded, Tries not to hurt others feelingsrecoded), and *unemotional* (5 items, e.g., Does not show emotions, It is easy to tell how he/she is feelingrecoded). For the present sample, internal consistency (Cronbach's **) is overall high for all three scales (*callousness *=0.77, *uncaring *=0.86; *unemotional *=0.77). For all further analyses, original ICU-scores were standardized using z-scores.

*Social-emotional competence and aggressive behavior.* Childrens level of emotional competence and aggressive behavior was assed at preschool age, using a German questionnaire, the *Behavior Rating Scales for Preschoolers* (Verhaltensskalen fr das Kindergartenalter, VSK; [33]) in a preschool teachers rating. For our analyses, we used the scales *emotional knowledge/empathy* (7 items, e.g., Reacts with concern when another child cries, Feels guilty when he/she has accidentally hurt others), *social competence* (6 items, e.g., Shares toys with other children, Invites other children to play), and *aggressive behavior* (10 items, e.g., Destroys objects, Insults others). Items were rated for the past four weeks on a four-level scale from "not true" (0) to "true" (3). All scales show high internal consistency for our studys sample (*emotion knowledge/empathy *=0.83; *social competence *=0.71; *aggressive behavior *=0.92). For all further analyses, original VSK-scores were standardized using z-scores.

### Data analytic strategy

In a first step, we aimed to examine the factor structure of CU traits in preschool aged children. Six alternative confirmatory factor analyses were conducted to find the best fitting model for our preschool sample by using the ICU items [15]. We first tested an undifferentiated model with a single factor (model 1). Based on the models of the ICU factor structure as reported in the existing literature, we also tested the following models:

- a two-factor model (model 2) with a *callousness* and an *uncaring* factor including 12 Items (c.f. [6]),
- a two-factor model (model 3) with a *callous-unemotional* and an *empathic/prosocial* factor (c.f. [25]),
- a three-factor model (model 4) with the factors *callousness, uncaring and unemotional* (c.f. [12]),
- a three-factor-higher-order hierarchical model (model 5) with the factors *general*, *callousness*, *uncaring* and *unemotional* (c.f. [16]),
- a three-factor-bi-factor model (model 6) with the factors *general*, *callousness*, *uncaring* and *unemotional* (c.f. [17, 18, 23]).

All models were run with the *Maximum-Likelihood* method with robust *Satorra-Bentler* estimation [34] to adjust for non-normality of the data. To evaluate the model fit, the common goodness-of-fit indices are reported including *Satorra-Bentler scaled chi-square statistic* (S-B **^*2*^), *Comparative Fit Index* (CFI), *Tucker-Lewis Index* (TLI), *Root-Mean-Square Error of Approximation* (RMSEA), *Standardized Root Mean Squared Residual* (SRMR), and *Akaikes Information criterion* (AIC). A non-significant *S-B *^*2*^ suggests good model fit as well as *CFI* and *TLI* values above 0.90, *RSMEA* and *SRMR* values below 0.08, and lower *AIC* values in model comparison [35,37].

Post hoc cluster analytic strategies were applied to identify groups of children with regard to their combination of social-emotional competences and CU traits. For this purpose, factors from the best fitting CFA model were used as CU variables (*callousness* and *uncaring*; as according to [6]). *Emotional knowledge/empathy* and *social competence* were used as social-emotional competence scales. All variables were included as *z*-scores.

We chose a combination of hierarchical and non-hierarchical (partitioning) clustering techniques. To identify outliers, we first used the hierarchical *single-linkage* method. After these were eliminated to obtain homogeneous clusters, the hierarchical *ward's-linkage* method was applied for cluster fusion and for determining the number of clusters. The *Duda-Hart* index as a reference measure proposed a solution with three clusters. For cluster optimization, in a next step, the non-hierarchical *k*-*means* method with *k*=3 clusters was used. In order to obtain three clusters, each observation was assigned to the cluster with the closest mean value. For all clustering methods (*single-linkage*, *ward's-linkage* and *k-means*), the *squared euclidean distance* was used as the distance measure.

By performing **^*2*^-test and ANOVA, significant group differences, as well as gender differences were tested for each cluster. In a last step, ANOVA with aggressive behavior as dependent variable was conducted to analyze group differences between the clusters. As the *Bartletts test* reveals partly unequal variances, the ANOVAs were estimated using the bootstrap method with 95% confidence intervals and a bootstrap 1000 samples [38].

## Results

### Factor structure of CU traits in preschool aged children

Six primary confirmatory factor analyses were tested to analyze the fit of the ICU items for our preschool sample. Whereas the single-factor model, the two-factor models, and the three-factor model converged, estimation problems occurred for the bi-factor-model and the higher-order hierarchical model. The model fit indices of the confirmatory factor analyses for the ICU Items are shown in Table 1.

**Table 1:** Model fit indices of the confirmatory factor analyses for the ICU Items (ML with S-B estimation)

Model	Description	*S-B *^*2*^_*(df)*_	*p*	*S-B CFI*	*S-B TLI*	*S-B RMSEA*	*SRMR*	*AIC*
1	One-factor (undifferentiated)	1244.252_(252)_	<.001	.662	.630	.103	.094	19,218.359
2	Two-factor (callousness, uncaring; 12 items)	117.388_(53)_	<.001	.946	.932	.048	.057	8891.095
3	Two-factor (callous-unemotional, empathic/prosocial; 24 items)	1126.912_(251)_	<.001	.702	.672	.090	.097	19,079.839
4	Three-factor (callousness, uncaring, unemotional; 24 items)	910.314_(249)_	<.001	.775	.751	.085	.080	18,835.897
5	Three-factor-higher-order-factor (general, callousness, uncaring, unemotional; 24 items)	No convergence						
6	Three-factor-bi-factor (general, callousness, uncaring, unemotional; 24 items)	No convergence						

*ML*Maximum Likelihood, *S-B*Satorra-Bentler, **^*2*^Chi-square statistic, *df*degrees of freedom, *CFI*Comparative Fit Index, *TLI*Tucker-Lewis Index, *RMSEA* Root Mean Square Error of Approximation, *SRMR*Standardized Root Mean Squared Residual, *AIC*Akaikes Information Criterion; *N*=371

For the converging models, the *S-B *^*2*^ reached significance. However, this is not surprising given the sample size of *N*>200 [39]. Comparing the converging models, model 2 reveals the best fit to the data (*CFI*=0.946, *TLI*=0.932, *RMSEA*=0.057, *SRMR*=0.048, *AIC*=8891.095). The model includes a callous and an uncaring factor using 12 of the original 24 ICU items (as according to [6]). Table 2 summarizes the included items and the associated path coefficients for model 2. All factor loadings in the 12 item two-factor solution show high significance with highly correlated factors (*r*=0.838; *p*<0.001).

**Table 2:** Path coefficients ICU model with latent factors callousness and uncaring

Item	Loadings	
	Callousness	Uncaring
ICU 04. Does not care who he/she hurts to get what he/she wants	.694***	
ICU 06. Does not show emotions	.419***	
ICU 09. Does not care if he/she is in trouble	.538***	
ICU 11. Does not care about doing things well	.175**	
ICU 12. Seems very cold and uncaring	.570***	
ICU 18. Shows no remorse when he/she has done something wrong	.675***	
ICU 21. The feelings of others are unimportant to him/her	.709***	
ICU 05. Feels bad or guilty when he/she has done something wrong^a^		.450***
ICU 08. Is concerned about the feelings of others^a^		.797***
ICU 16. Apologizes (says he/she is sorry) to persons he/she has hurt^a^		.771***
ICU 17. Tries not to hurt others feelings^a^		.854***
ICU 24. Does things to make others feel good^a^		.560***

^a^Reverse-scored items; *p<.05; **p<.01; ***p<.001

### Social-emotional competencies and CU traits in preschoolers

In a next step, we conducted cluster analytic strategies to identify groups of preschoolers concerning their social-emotional competencies and CU traits. Based on the CFA analyses, we included CU traits by computing the items from the best fitting CFA model into the scales callousness and uncaring (see Table 2). Due to a low factor loading below 0.4, ICU item 11 (Does not care about doing things well) was excluded for further analyses.

**Fig. 1:**
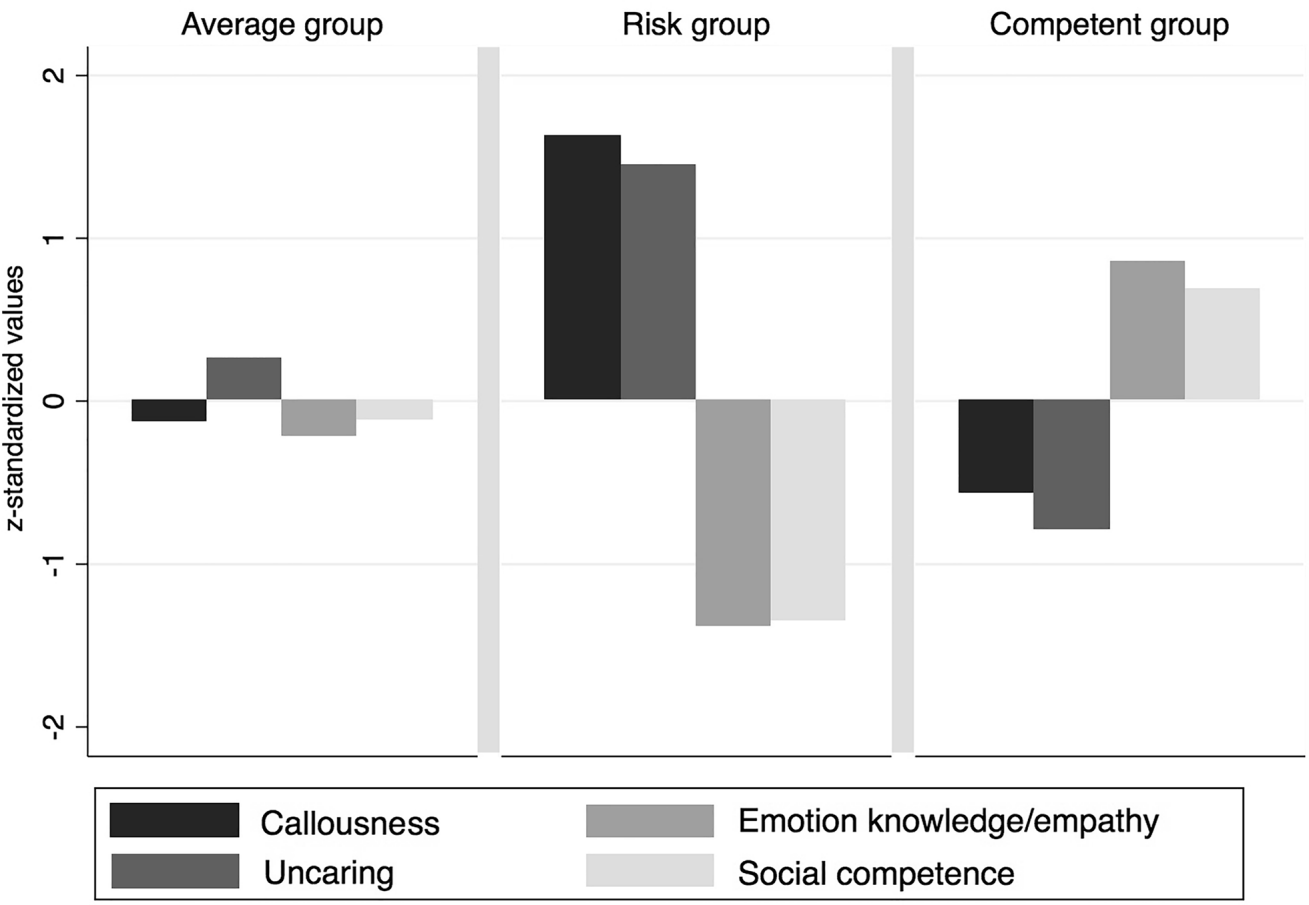
Means of clustered variables corresponding to the identified groups of preschool children (z-standardized values)

Table 3 summarizes the descriptive statistics and intercorrelations among the cluster variables. Graphical dendrogram analysis of the *single-linkage* clustering revealed seven outliers, which were eliminated for further proceedings. The *Duda-Hart* index after *wards-linkage* clustering supported a three-cluster solution. Thus, the *k*-*means* method with *k*=3 clusters was applied for cluster optimization. The results are shown in Fig. 1.

**Table 3:** Descriptive statistics and intercorrelations among the cluster variables and aggressive behavior

									Range	
	Variable	1	2	3	4	5	*M*	*SD*	Minimum	Maximum
1	Callousness^a^	1					2.32	2.85	0	12
2	Uncaring^a^	.674***	1				6.02	3.25	0	15
3	Emotion knowledge/empathy^b^	.655***	.752***	1			14.37	4.25	1	21
4	Social competence^b^	.549***	.584***	.626***	1		14.51	2.75	6	18
5	Aggressive behavior^b^	.696***	.695***	.582***	.446***	1	7.38	7.04	0	27

*M*mean, *SD*standard deviation

^a^ICU as according to Hawes et al. [6], ^b^scales from the *Behavior Rating Scales for Preschoolers* [33],

*p<.05; **p<.01; ***p<.001

The clusters can be described as an average group (*n*=140; 38.46%), a risk group (*n*=60; 16.48%), and a competent group (*n*=164; 45.05%). The competent group includes children with high emotional knowledge/empathy and social competence, and low callousness and uncaring. The risk group demonstrates high rates of callousness and uncaring combined with weak emotional knowledge/empathy and social competence. The average group involves children who show average characteristics in all cluster variables. The descriptive statistics for all clusters and cluster variables are presented in Table 4. The risk group includes significantly more boys (**^*2*^=19.748, *p*<0.001), while the competent group and the average group include significantly more girls (competent group **^*2*^=28.792, *p*<0.001; average group **^*2*^=4.084, *p*<0.05).

**Table 4:** Descriptive statistics for the clustered variables (z-standardized values)

	Competent group	Risk group	Average group
	*n* (%)	*n* (%)	*n* (%)
Total sample	164 (45.05%)	60 (16.48%)	140 (38.46%)
Girls	107 (59.12%)	14 (7.73%)	60 (33.15%)
Boys	57 (31.15%)	46 (25.14%)	80 (43.72%)

	M (SD)	M (SD)	M (SD)
Callousness			
Total sample	.56 (.03)	1.63 (.09)	-.12 (.05)
Girls	57 (.03)	1.78 (.26)	-.18 (.09)
Boys	.55 (.05)	1.59 (.10)	-.08 (.07)
Uncaring			
Total sample	.79 (.05)	1.45 (.08)	.28 (.05)
Girls	.78 (.06)	1.43 (.15)	.23 (.08)
Boys	.80 (.08)	1.46 (.10)	.29 (.06)
Emotion knowledge/empathy			
Total sample	.85 (.04)	-1.38 (.08)	-.22 (.04)
Girls	.95 (.04)	-1.21 (.18)	-.15 (.05)
Boys	.66 (.06)	-1.43 (.09)	-.26 (.06)
Social competence			
Total sample	.68 (.04)	-1.35 (.09)	-.12 (.06)
Girls	.69 (.06)	-1.73 (.13)	-.21 (.08)
Boys	.68 (.08)	-1.23 (.10)	-.05 (.08)

*M *mean, *SD*standard deviation; *N*=371

### Associations with aggressive behavior

The bootstrapped ANOVAs revealed significant group differences for all cluster variables per cluster (see Table 5). The children in the three clusters differ significantly in their level of aggressive behavior (bootstrapped *F*=160.735, *SE*=28.817,* p*<0.001, CL [104.256217.215]). Children in the risk group demonstrate the highest levels of aggressive behavior (*z*-score *M*=1.34, *SE*=0.11) while children in the competent group show the lowest levels of aggressive behavior (*z*-score *M*= 0.61, *SE*=0.04). Children in the average group show average levels of aggressive behavior (*z*-score *M*=0.07, *SE*=0.07).

**Table 5:** Group differences for cluster variables and aggressive behavior per cluster (average group, risk group, competent group)

	*F*	*SE*	*p*	95% CI
Callousness	326.688	52.864	0.000	[223.077430.299]
Uncaring	353.237	42.2447	0.000	[270.439436.035]
Emotion knowledge/empathy	483.633	62.4633	0.000	[361.208606.059]
Social competence	225.600	32.5885	0.000	[161.728289.473]
Aggressive behavior	160.735	*SE*=28.817	0.001	[104.256217.215]

*SE*standard error, CIconfidence interval, bootstrapped coefficients and confidence intervals; bootstrap of 1000 samples; *N*=371

## Discussion

Although the ICU's factor structure and procedures have been criticized before [24, 40], the questionnaire has often been used without scrutiny. Literature indicates inconsistencies in the specification of the construct of CU traits using the ICU in samples of children and adolescents. Factor-analytic studies involving younger children at preschool age are especially rare. This is problematic, as even tough associations between CU traits and developmental trajectories have been replicated in a number of samples (e.g., [6, 12, [25]), these studies often do not consider childrens social-emotional development in their analyses. However, not considering childrens social-emotional development when investigating this relationship, especially at a young age, leads to a limited interpretation of results.

Our study focuses on conceptualizing CU traits in preschoolers and is one of the first to analyze the factor structure of the ICU for a preschool sample. We examined several models of the ICU that were extracted from the literature on our data. A two-factor model including 12 of the original 24 ICU items, comprising of the factors callousness and uncaring, revealed the best fit to our data. This replicates the findings by Hawes et al. [6]. Similar results were found by Bansal et al. [24] who also tested existing ICU models in a sample of 104 children.

The concept of three ICU subscales (callousness, uncaring, unemotional) according to Frick [15] was therefore not supported by our data. Weakness in the scale *unemotional* has already been pointed out in a meta-analytical study of Cardinale and Marsh [41]. Besides methodological reasons (based on item pooling and wording) and smaller correlations with an overarching CU factor, the unemotional scale may not be a strong predictor of externalizing outcomes as the other two scales: callousness and uncaring [16, 41, 42].

Complex models reported for youth and adolescent samples could not be replicated for our preschool sample. The most evaluated three-factor-bi-factor model [17, 18, 23] did not find convergence. In previous studies, the three-factor-bi-factor model was primarily supported in samples of older children / adolescents, which indicates that established models cannot simply be transferred to different age groups. Especially in childhood, a differentiated measurement of CU traits is hampered if other developmental factors are not incorporated.

Therefore, in a second step, we aimed to identify groups of preschool children with regard to both CU traits and their social-emotional development in order to investigate associations with levels of aggressive behavior. Since CU traits are characterized by features of social-emotional competencies or a lack of competencies respectively, such as lack of empathy, low emotional responsiveness and unconcern about others [29], it is difficult to distinguish CU traits and social-emotional competencies in preschool children.

The knowledge of emotions and managing them is crucial for positive social interactions and the development of stable relationships [43]. Thus, emotional competencies are important for the acquisition of social skills and especially for the development of prosocial behavior and empathy [44]. Social and emotional competence in children reduces their involvement in aggressive interactions [45, 46]. However, it needs to be considered that the social-emotional development at preschool age is still in progress.

In our sample, we identified three groups of preschool children regarding their level of CU traits and social-emotional competencies. A competent group includes children with high emotional knowledge/empathy and social competence, while showing low levels of callousness and uncaring. A risk group comprises of children with high rates of callousness and uncaring combined with weak emotional knowledge/empathy and social competence. The average group involves children who show average characteristics in all cluster variables. All preschool clusters differ significantly in their level of aggressive behavior, with the risk group showing the highest mean of aggressive behavior compared to competent group and the average group. We were therefore able to replicate the relationship between high CU traits and behavioral problems such as aggressive behavior, as shown in a number of previous studies (e.g., [6, 9, 12, 20, 25]).

## Limitations and further directions

The results of the present study should be considered alongside several limitations. One of our goal was to analyze the factor structure of CU traits in preschool children. However, for this purpose, we only tested existing confirmatory models of the ICU and neglected an explorative analysis. Of the six CFA models, only one model (two-factor model with reduced items according to [6] fits the data of our preschool sample well. Potentially, the ICU or all ICU items respectively may not be adequate to assess CU traits in young children in a developmentally appropriate manner. In continuing research, different approaches should be used to further conceptualize CU traits in childhood. Besides exploratory analysis, item response theory techniques can be applied to identify psychometrically suitable ICU items for a preschool sample (similar to the procedures of [6]. This should be followed by a joint analysis of CU items and social-emotional competence items to address the issue that CU items are commonly formulated as (missing) social-emotional competencies (compare [25]. However, at preschool age, childrens social-emotional development is still processing, which calls into question the differentiation of CU traits.

A further limitation concerns the analysis of cross-sectional data. In childhood, many behavioral features are not rigid, but underlie a progressive development process. Studies need to address this process in their analyses to achieve meaningful and reliable results. Not only CU variables should be assessed over several timepoints of measurement, but also the development of aggressive behavior as a potential outcome.

## Conclusions

The current results advance the past work in showing that these associations with aggressive behavior especially exist for children, who demonstrate high levels of CU traits and at the same time, low levels of social-emotional competencies. Therefore, characteristics of the competent group and the risk group indicate that the construct of CU traits in childhood may be nothing other than a social-emotional developmental deficit. High rates of CU traits are associated with low social-emotional competencies (emotional knowledge/empathy and social competence) and vice versa. These results may lead to the conclusion that CU traits and social-emotional competencies in childhood cannot be separated from each other, but can rather be described as a continuum. Social-emotional impairments seem to go hand in hand with callousness and uncaring behaviors.

On the other hand, we found an average group of preschoolers who demonstrate an average level of CU behavior and social-emotional competencies. At this point it remains unclear whether this group exemplifies the fact that CU traits and social-emotional competencies are two independent constructs or whether the children in this average group are still in the middle of their social-emotional developmental process.

We also found gender differences for the clusters. The risk group includes significantly more boys while the competent group and the average group include significantly more girls. Other studies have repeatedly shown that boys in particular show increased CU behavior [12, 16]. Following the approach that CU traits in childhood can be explained as a social-emotional developmental deficit, gender differences can be explained by the fact that girls are generally more advanced in their social-emotional development compared to boys (c.f. [47, 48]. Conclusively, our results indicate that boys with low social-emotional competencies combined with high callousness und uncaring features are at the greatest risk of exhibiting advanced aggressive behaviors.

Our study is one of the first to focus on the conceptualization of CU traits in preschool children, taking into account social-emotional developmental variables. The findings lead the question of whether CU traits or rather a lack of social-emotional skills are potential early markers of psychopathy in young children.

## Data Availability

The datasets analysed in the current study are available from the corresponding author on reasonable request.
